# Iron metabolism mediates microglia susceptibility in ferroptosis

**DOI:** 10.3389/fncel.2022.995084

**Published:** 2022-08-30

**Authors:** Lingling Jiao, Xiaolan Li, Yuxiang Luo, Junfen Wei, Xulong Ding, Huan Xiong, Xuesong Liu, Peng Lei

**Affiliations:** ^1^Department of Neurology and State Key Laboratory of Biotherapy, West China Hospital, Sichuan University, Chengdu, China; ^2^Department of Neurosurgery, West China Hospital, Sichuan University, Chengdu, China

**Keywords:** cell death, ferroptosis, iron metabolism, glia, neurons

## Abstract

Ferroptosis is implicated in a range of brain disorders, but it is unknown whether neurons or glia in the brain are particularly effected. Here, we report that primary cortical astrocytes (PA), microglia (PM), and neurons (PN) varied in their sensitivities to ferroptosis. Specifically, PM were the most sensitive to ferroptosis, while PN were relatively insensitive. In contrast, PN and PM were equally susceptible to apoptosis, with PA being less affected, whereas all three cell types were similarly susceptible to autophagic cell death. In the tri-culture system containing PA, PM, and PN, the cells were more resistant to ferroptosis than that in the monoculture. These results demonstrated that brain cells exhibit different sensitivities under ferroptosis stress and the difference may be explained by the differentially regulated iron metabolism and the ability to handle iron. Continued elucidation of the cell death patterns of neurons and glia will provide a theoretical basis for related strategies to inhibit the death of brain cells.

## Introduction

Neurological diseases significantly impact the life of the elderly. However, many of these diseases, including Alzheimer’s disease (AD) and multiple sclerosis (MS), still lack effective therapeutic interventions. Brain iron accumulation in the affected regions and increased oxidative stress have been noted in various neurological disorders (Hare et al., 2013), and chelation of iron has been suggested as a therapeutic strategy (Devos et al., 2014; Moreau et al., 2018; Klopstock et al., 2019). Recently, ferroptosis, a regulated cell death driven by iron-dependent phospholipid peroxidation, has also been implicated in several neurological disorders, such as AD, Parkinson’s disease (PD), MS, ischemic and hemorrhagic stroke (Do Van et al., 2016; Tuo et al., 2017; Bao et al., 2021; Lei et al., 2021; Yan et al., 2021; Luoqian et al., 2022; Tuo et al., 2022). Anti-ferroptosis compound has been trialed in phase I clinical studies for Amyotrophic Lateral Sclerosis (ALS) and PD, and the results warrant the next stage of clinical investigations (Southon et al., 2020). Therefore, ferroptosis may be essential in the pathogenesis of neurological diseases.

Ferroptosis is regulated by multiple cellular metabolic pathways, including redox homeostasis, iron metabolism, mitochondrial activity, and lipids metabolism (Dixon et al., 2012; Yan et al., 2020; Yan et al., 2021). Glutathione peroxidase 4 (Gpx4) and ferroptosis suppressor protein 1 (FSP1) are two crucial check-points to prevent ferroptosis, through the antioxidant systems by catalyzing the reduction of lipid peroxides and regeneration of ubiquinone, respectively (Friedmann Angeli et al., 2014; Yang et al., 2014; Bersuker et al., 2019; Doll et al., 2019). On the other hand, acyl-CoA synthetase long-chain family member 4 (Acsl4) is crucial for the execution of ferroptosis through its catalytic role in polyunsaturated fatty acids production (Doll et al., 2017). In addition, proteins involved in iron regulation, including transferrin (Tf), transferrin receptor 1 (TfR1), divalent metal transporter 1 (DMT1), ferroportin (Fpn), ferritin heavy chain 1 (FTH1), iron-responsive protein 1/2 (IRP1/2), and hypoxia-inducible factor 1α (HIF-1α), have been implicated independently or synergistically in ferroptosis (Masaldan et al., 2019; Tian et al., 2020; Bao et al., 2021).

Cell death occurs extensively in both neurons and glial cells during neurological diseases. Neuronal death caused by ferroptosis has been reported in neurological diseases such as AD, MS, and stroke (Bao et al., 2021; Luoqian et al., 2022; Tuo et al., 2022). The glial cells, including astrocytes and microglia, are considered dynamic guardians of neurons, and function to maintain the blood-brain-barrier, modulate synapse activity and respond to central nervous system (CNS) injuries (Prinz et al., 2019; Sofroniew, 2020). It has been reported that ferroptosis also occurred in astrocytes and microglia during neurodegenerative conditions, and inhibition of its activation in astrocytes can prevent neuronal death (Ishii et al., 2019; Kapralov et al., 2020; Park et al., 2021). Different cell lineages exhibit differentiated susceptibility to ferroptosis in cancer cell lines (Yang et al., 2014; Zou et al., 2020), but the sensitivity of specified brain cells in response to ferroptosis has yet to be explored.

Here, we have isolated and cultured primary cortical astrocytes (PA), microglia (PM), and neurons (PN) to compare their sensitivities to ferroptosis and other types of cell death such as apoptosis and autophagy. We have then applied a tri-culture system to mimic ferroptosis phenomena *in vivo*, and to determine the sequence of cell death in different cells. We have also explored the potential mechanisms to explain different sensitivities by examining the changes in ferroptotic genes as well as iron-regulatory genes. These results may provide a theoretical basis for inhibiting ferroptosis in specific brain cell types in neurological diseases.

## Materials and methods

### Animals

All animal care and experimental protocols were approved and performed following the guide from the Institutional Guidelines of the Animal Care and Use Committee (K2018071, Sichuan University, China). Timed-pregnant Sprague-Dawley rats were purchased from the Institute of Laboratory Animals of Sichuan Academy of Medical Sciences, China. Primary cortical cell cultures were all prepared from postnatal day 0–1 rat pups. All animals were housed under standard conditions of temperature (22 ± 2°C) and humidity, and a 12 h light-dark cycle, with free access to food and water before experiments.

### Primary culture preparation

Primary cortical cell cultures were all prepared from postnatal day 0–1 rats (Hennebelle et al., 2020). The plates or flasks were pre-coated with 1 mg/mL of poly-D-lysine (Sigma, United States) overnight at 37°C with 5% CO_2_, then washed with sterile deionized water and covered with the corresponding medium detailed below.

PN from the cortices was prepared as previously described (Lei et al., 2012). Cortices were dissected and dissociated in trypsin (Sigma, T-4665, United States). The neurons were plated at a density of 6*10^∧^5/mL in the plating medium (DMEM with 10% fetal bovine serum, 5% horse serum, and 10 mg/L gentamycin sulfate, purchased from Invitrogen or Sigma, United States). After 2 h incubation, the neurons were changed to Neurobasal supplemented medium (with B27, 500 μM glutaMAX and 10 μg/mL gentamycin sulfate, Thermo Fisher Scientific or Sigma, United States). The experiments were performed on day 7 post-plating.

PA and PM were also prepared from postnatal day 0–1 rat cortices as previously described (Xu et al., 2019). In brief, the brain tissues of rat pups were dissected, and then mechanically dissociated and subsequently strained through a 70 μm cell strainer (BD, United States) until full dissociation into a single-cell suspension. After centrifugation at 1,000 rpm for 5 min, the pellets were resuspended in a complete medium (DMEM/F12 1% penicillin/streptomycin and 20% fetal bovine serum, Thermo Fisher Scientific, United States) and plated in poly-D-lysine-coated 150-cm^2^ flasks. After 7–14 days, the confluent culture was placed horizontally on a shaker (Quanterix, United States) with the medium covering the flasks and shaken at 250 rpm for 16–18 h. The supernatant detached cells were collected as PM. The attached cells were trypsinized and then collected as PA. Both PA and PM were plated on poly-D-lysine coated 6-well plates 2 days before experiments.

### Primary cortical co/tri-culture systems preparation

The tri-culture system was established following a protocol modified from previous work (Goshi et al., 2020). In this study, the cell slides were coated with poly-D-lysine before plating. PN were cultured on the cell slides for 5 days before tri-culture, then the collected PA and PM were plated separately into the cell slides which were covered with PN for 2 days before experiments. In the co-culture system (PA and PN), the steps are the same as in the tri-culture. The workflow was shown in Figure 4A.

### Cell viability assay

Primary cells were plated onto 96-well plates and treated with Ferrous ammonium sulfate hexahydrate (H_20_FeN_2_O_14_S_2_, Fe^2+^) (0–1 mM in ddH_2_O; Sigma, United States, 7783-85-9) or different cell death inducers (RSL3, 0–100 μM in DMSO, S8155; erastin in DMSO, 0–100 μM, S7242; Selleck Chemicals, CN) to induce ferroptosis (Dixon et al., 2012; Tuo et al., 2022), staurosporine (0–100 μM in DMSO; Selleck Chemicals, CN, S1421) to induce apoptosis (Tang et al., 2000), and rapamycin (0–100 μM in DMSO; Selleck Chemicals, CN, S1039) to induce autophagy (He et al., 2017) after seeding. Cell viability was assayed 24 h post-treatment using Cell Counting Kit-8 (CCK8, Bimake, United States) at the optical density of 450 nm or lactate dehydrogenase (LDH) Cytotoxicity Assay Kit (Thermo Fisher Scientific, United States) following the manufacturer’s instructions at the optical density of 490 nm. The data were normalized against the control.

### Flow cytometry analysis for lipid reactive oxygen species detection

The lipid reactive oxygen species (ROS) within cells were detected using a flow cytometry method as previously described (Tuo et al., 2022). Briefly, primary cells were collected and washed with phosphate buffer saline (PBS, Thermo Fisher Scientific, United States), then the cells were incubated with 1 μM BODIPY 581/591 C11 (D3861, Thermo Fisher Scientific, United States) for 30 min at 37°C in an incubator (Thermo Fisher Scientific, United States). Subsequently, cells were resuspended in 500 μL hank’s balanced salt solution (Thermo Fisher Scientific, United States), strained through a 70 μm cell strainer (BD, United States), and analyzed using the 488 nm laser of a flow cytometer (LSR Fortessa, BD, United States). For BODIPY 581/591 C11 staining, the signals from both non-oxidized C11 (PE channel) and oxidized C11 (FITC channel) were monitored. The ratios of mean fluorescence intensity (MFI) of FITC to that of PE were calculated. Data analysis was conducted using FlowJo X software (BD, United States).

### Immunocytochemistry staining

Cells were fixed with 4% paraformaldehyde (PFA, Sigma, United States) at room temperature for 30 min. Then, cells were blocked for 1 h at room temperature with 6% normal goat serum (Solarbio, CN) dissolved in 0.2% Triton X-100 and incubated with specific primary antibodies overnight at 4°C in a humidified chamber. The antibodies used in the study were: primary antibodies TuJ-1 (1:10,000, Sigma, United States, T2200), glial fibrillary acidic protein (GFAP), (1:1,000 Millipore, United States, MAB360), ionized calcium-binding adaptor molecule 1 (Iba-1) (1:2,000, Wako, JPN, 019-19741). For the negative control, the primary antibody was replaced by 0.01 M PBS. The next day, cells were washed three times for 10 min each by 0.01 M PBS and incubated with the secondary antibody of Alexa Fluor^®^ 488 AffiniPure Alpaca Anti-Rabbit IgG (H + L) (Jackson ImmunoResearch, United States, 611-545-215) or Cy™3 AffiniPure Alpaca Anti-Rabbit IgG (H + L) (Jackson ImmunoResearch, United States, 611-165-215) for 1 h at room temperature. After three times washing for 10 min each with 0.01 M PBS, nuclei were stained with DAPI (Beyotime, CN) for 10 min. Images were taken under a fluorescent microscope (OLYMPUS VS200, JPN) equipped with a digital camera and the OlyVIA software (OLYMPUS, Ver.3.3, JPN). At least four non-overlapping images were automatically counted by Image J (1.49 m, NIH, United States) in a double-blinded manner.

### Quantitative real-time polymerase chain reaction

Cells were collected for the investigation of mRNA expression by quantitative real-time polymerase chain reaction (qPCR). Total RNA was extracted using the TRIzol reagent (Invitrogen, United States) by the manufacturer’s protocol and was quantified by spectrophotometry (NanoDrop 2000 Fluorospectrometer, Thermo Fisher Scientific, United States). RNAs were reversely transcribed to complementary DNA (cDNA) by a reverse transcriptase kit (Thermo Fisher Scientific, United States). The relative abundance of each mRNA was quantified by qPCR using specific primers and the TransStart^®^ Tip Green qPCR SuperMix (Transgene, CN). Primers for rats were synthesized by YouKang (CN) and listed in Table 1. qPCR reactions were carried out using Real-Time PCR Detection System (C1000 Touch™ Thermal Cycler, Bio-Rad, United States). Data were analyzed by the 2^–ΔΔCt^ method and normalized against *Gapdh*.

**TABLE 1:** PCR primers sequences.

Primer	Sequence
Acsl4-F	TCTGAGCAACAGCGAAGGTT
Acsl4-R	GAATTAGCAGCACCCGACCT
Aifm2-F	CCTTGAGATAGCAGCCTCCG
Aifm2-R	CCAACAAAGGGTGAGCTTGC
Dmt1-F	GGCTAATGGTGGAGTTGG
Dmt1-R	CTGCGATGGTGATGAGG
Fpn-F	TGGGTGGATAAGAATGCC
Fpn-R	ATGATCCCGCAGAGAATG
Gapdh-F	AGTGCCAGCCTCGTCTCATA
Gapdh-R	GGTAACCAGGCGTCCGATAC
Gpx4-F	CCTGGCTGGCACCATGT
Gpx4-R	TATCGGGCATGCAGATCGAC
Hif-1α-F	ATGCCAGATCACAGCACA
Hif-1α-R	GGACAAACTCCCTCACCA
Irp1-F	TTCACTCTTGCTCATGGCT
Irp1-R	CGCCTCTACGGCTTTCT
Irp2-F	GAACTGGAATGGCTCATCA
Irp2-R	CAATTACGCTGTCTGGGAA
Tf-F	CTGCCATTCGAAATCAGCGG
Tf-R	TGACGCTCCACTCATCACAC
Tfr-F	GGCTTCCCAACATCCCT
Tfr-R	GCGACCCTTCCATTCCT

### Statistical analysis

All data are shown as the mean ± SEM. The Student’s *t*-test was used to detect differences between two groups; for multiple comparisons, one-way ANOVA with a *post hoc* Tukey test was performed using GraphPad Prism 8.0 software. *P* < 0.05 was considered statistically significant.

## Results

### Primary microglia were most susceptible to ferroptosis

**FIGURE 1:**
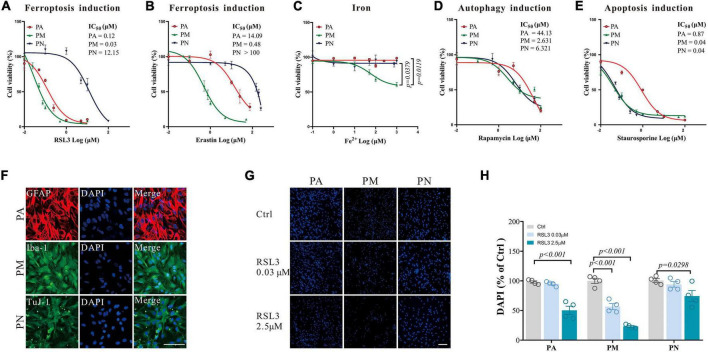
The response of primary brain cells to cell death inducers. **(A–E)** Dose-response curves of ferroptosis-inducers RSL3 **(A)** or erastin **(B)**, Fe^2+^ **(C)**, autophagy-inducer rapamycin **(D)**, and apoptosis-inducer staurosporine **(E)** on primary brain cells after 24 h treatment, detected by CCK8, respectively *N* = 6. **(F)** Brain cells in the monoculture systems were stained with Iba-1 (green), TuJ-1 (green), GFAP (red), and DAPI (blue), respectively. **(G)** Brain cells in the monoculture systems were stained with DAPI (blue) after RSL3 treatment for 24 h, respectively. **(H)** The quantification of the proportion of DAPI in primary brain cells in the monoculture systems. Data are means ± SEM, *N* = 4, scale bar = 100 μm. One-way ANOVA with *post hoc* Tukey test was performed.

To examine the responses to ferroptosis in different brain cells, primary cells were isolated and cell death inducers were applied to these cells. PA, PM, and PN show highly differentiated responses to either RSL3 (IC_50_ (μM): PA = 0.12, PM = 0.03, PN = 12.15, Figure 1A) or erastin (IC_50_ (μM): PA = 14.09, PM = 0.48, PN > 100, Figure 1B) induced ferroptosis, where PM were the most sensitive to ferroptosis and PN were the least sensitive (Figures 1A,B). These observations were consistent with the sensitivities of these cells to iron toxicity, where PM have shown significant cell death at concentrations where PA and PN were viable (Figure 1C). These results have been further validated by immunostaining using GFAP, Iba-1, TuJ-1 and DAPI to label PA, PM, PN and their nuclei, respectively (Figures 1F–H). Meanwhile, staurosporine-induced apoptosis and rapamycin-induced autophagy were applied in these primary cells. Staurosporine induced significantly less cell death in PA compared to PM and PN (IC_50_ (μM): PA = 0.87, PM = 0.04, PN = 0.04, Figure 1D), where rapamycin resulted in similar levels of cell death among tested cells (IC_50_ (μM): PA > 100, PM = 7.471, PN = 46.96, Figure 1E). These results suggest that different types of brain cells respond to cell death signaling differently, and PM were the most sensitive cell type tested to ferroptosis and iron stress.

**FIGURE 2:**
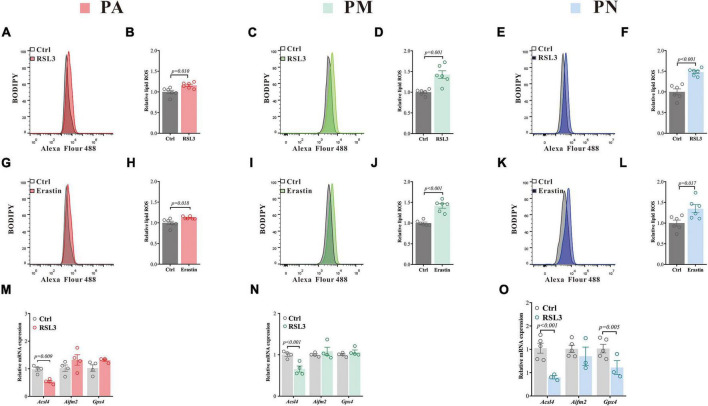
Ferroptosis inducers lead to increased ROS and changes in ferroptosis-related gene expressions in primary brain cells. **(A–F)** Lipid ROS in PA **(A)**, PM **(C)** and PN **(E)** treated with RSL3 for 24 h (representative histogram plot for fluorescence of oxidized BODIPY-C11), respectively. Relative lipid ROS is presented as the ratio of oxidized to reduce BODIPY-C11 MFI in PA **(B)**, PM **(D)** and PN **(F)** treated with RSL3 for 24 h, respectively *N* = 6. **(G–L)** Lipid ROS in PA **(G)**, PM **(I)** and PN **(K)** treated with erastin for 24 h (representative histogram plot for fluorescence of oxidized BODIPY-C11), respectively. Relative lipid ROS is presented as the ratio of oxidized to reduce BODIPY-C11 MFI in PA **(H)**, PM **(J)** and PN **(L)** treated with erastin for 24 h, respectively *N* = 6. **(M,N)** qPCR was performed to quantify the mRNA levels of ferroptosis-related genes in PA **(M)**, PM **(N)** and PN **(O)** after 24 h of RSL3 treatment, respectively. Data are means ± SEM, *N* = 3–5. Student’s *t*-test was performed.

We have further measured biochemical features consistent with the occurrence of ferroptosis. Lipid peroxidation, measured by the BODIPY-C11 method, is considered one marker for ferroptosis (Doll et al., 2017). We have found that both RSL3 [(μM): PA = 0.08, PM = 0.02, PN = 2.5] and erastin [(μM): PA = 10, PM = 0.5, PN = 15] treatment (doses to induce about 20% of cell death in each cell type, respectively) caused a significant elevation of the lipid ROS in all cell types (Figures 2A–L). *Acsl4*, *Aifm2* (encoding FSP1), and *Gpx4* are crucial in ferroptosis (Yan et al., 2021), and RSL3 treatment significantly reduced the level of *Acsl4* in all tested cells, with an additional reduction of *Gpx4* in PN (Figures 2M–O). *Aifm2* was not affected in all cell types (Figures 2M–O). RSL3 is a Gpx4 inhibitor, which blocks the antioxidant activity of Gpx4 through direct binding (Yang et al., 2014), and usually without affecting its expression. The reduction of *Acsl4* we observed here may be a protective mechanism against lipid ROS accumulation, which was reported previously (Tuo et al., 2022).

### Differences in iron regulation may be responsible for ferroptosis sensitivity

**FIGURE 3:**
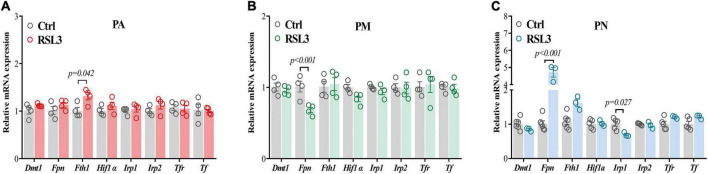
Iron metabolism-related gene expressions in primary brain cells after RSL3 treatment. **(A–C)** qPCR was performed to quantify the mRNA levels of iron metabolism-related genes in PA **(A)**, PM **(B)** and PN **(C)** after 24 h of RSL3 treatment, respectively. Data are means ± SEM, *N* = 3–5. Student’s *t*-test was performed.

PM were not only the most sensitive cells to ferroptosis but to iron as we have shown in Figures 1A–C. Since the regulation of ferroptosis cannot explain the differentiated sensitivities, we have hypothesized that iron metabolism may be involved since it has also been implicated in ferroptosis regulations (Lei et al., 2021). We have therefore examined the changes in iron regulatory genes in three brain cells treated with RSL3 by qPCR. We have used the same RSL3 doses as Figure 2, and found an increased level of *Fth1* only in PA (Figure 3A). A significant reduction of *Fpn* was observed in PM (Figure 3B), while it was up-regulated in PN (Figure 3C). The level of *Irp1* was also down-regulated in PN post RSL3 treatment (Figure 3C). It is known that glial cells regulate iron in a different way vs. neurons (Reinert et al., 2019) and the significantly elevated *Fpn* in PN after RSL3 treatment may facilitate iron exportation, which protects the neurons from ferroptosis or iron toxicity.

**FIGURE 4:**
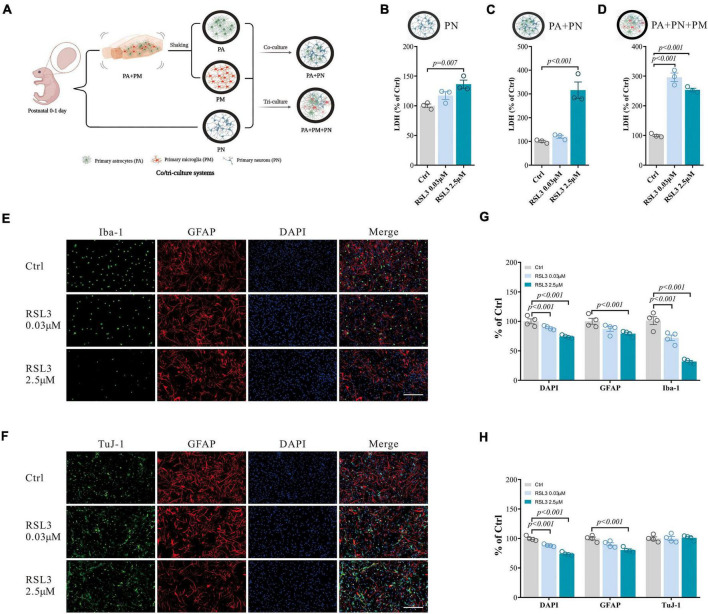
RSL3 induced primary brain cell death in co/tri-culture system. **(A)** Workflow for the co/tri-culture system (This image was created by BioRender.com). **(B–D)** Cell viability detected by LDH in PN monoculture **(B)**, PA/PN co-culture **(C)**, and PA/PN/PM tri-culture system **(D)** after RSL3 treatment for 24 h. **(E,F)** Brain cells in the tri-culture system were stained with Iba-1 (green), TuJ-1 (green), GFAP (red) and DAPI (blue) after RSL3 treatment for 24 h. **(G,H)** The quantification of the proportion of DAPI, GFAP, Iba-1, and TuJ-1 positive primary brain cells in the tri-culture system. Data are means ± SEM, *N* = 4, scale bar = 200 μm. One-way ANOVA with *post hoc* Tukey test was performed.

### Glia are more sensitive to ferroptosis in the tri-culture system

The monoculture system we used can only mimic brains with limited capacity. We have then tested the PA, PM, and PN responses to RSL3 (0.03 μM to induce about 50% PM death and 2.5 μM to induce 20% PN death according to Figure 1A) or erastin (0.5 μM to induce about 50% PM death and 15 μM to induce 20% of PN death according to Figure 1B) in co/tri-culture systems (1:1 or 1:1:1) modeling the *in vivo* environment (Figure 4A). We found that both PN/PA co-culture and PN/PA/PM tri-culture responded significantly to RSL3 treatment, while PN alone was less affected by the treatment which is consistent with our earlier results (Figures 4B–D). Consistent with the monoculture, immunostaining of tri-culture revealed that the DAPI and Iba-1 double-positive cells were significantly reduced dose-dependently with RSL3 treatment (Figures 4E,G), indicating that PM were affected most severely. GFAP-positive cells were reduced by treating with 2.5 μM RSL3 in the tri-culture system (Figures 4E–H), indicating the loss of PA similar to our previous observations in monoculture (Figures 1F–H). There were no significant changes in TuJ-1-positive cells (Figures 4F,H), indicating that neurons were less affected with RSL3 treatment in the tri-culture system.

**FIGURE 5:**
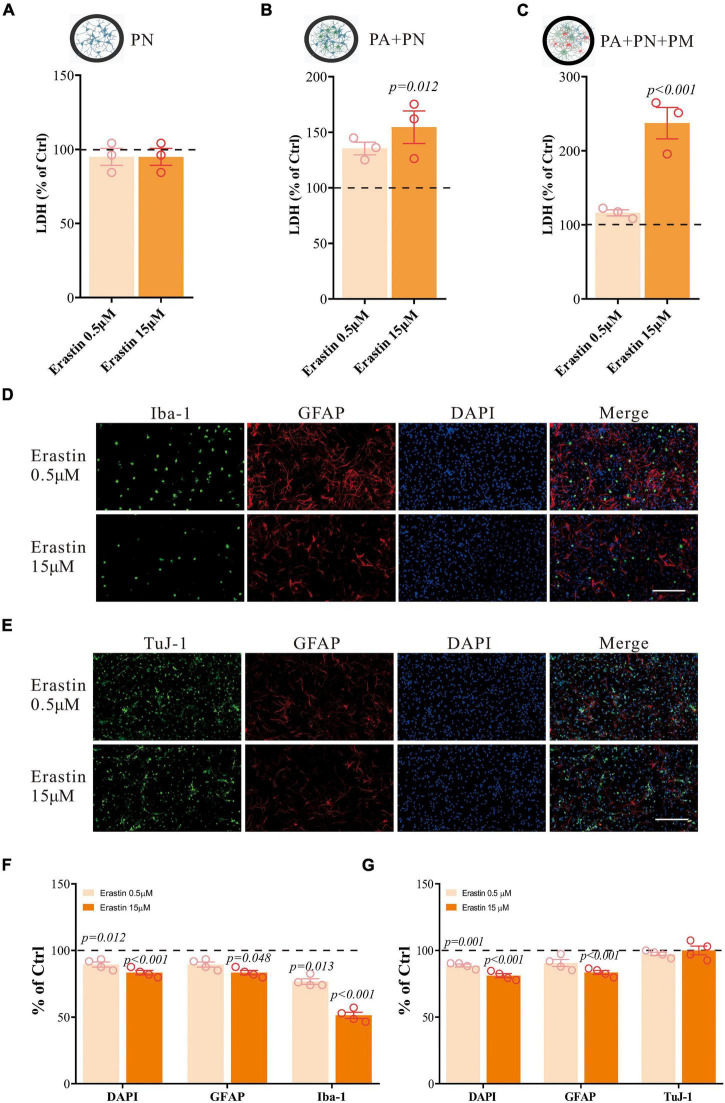
Erastin induces primary brain cell death in co/tri-culture system. **(A–C)** Cell viability detected by LDH in PN monoculture **(A)**, PA/PN co-culture **(B)**, and PA/PN/PM tri-culture system **(C)** after erastin treatment for 24 h. **(D–G)** Brain cells in the tri-culture system were stained with Iba-1 (green), TuJ-1 (green), GFAP (red) and DAPI (blue) after erastin treatment for 24 h **(D,E)**. The quantification of the proportion of DAPI, GFAP, Iba-1 and TuJ-1 positive cells in the tri-culture system **(F,G)**. Data are means ± SEM, *N* = 4, scale bar = 200 μm. One-way ANOVA with *post hoc* Tukey test was performed.

We have further validated our findings using erastin to induce ferroptosis. Erastin treatment in the tri-culture system also resulted in similar LDH changes as RSL3 treatment (Figures 5A–C). It resulted in a significant reduction of Iba1-positive cells, and a moderate reduction in GFAP-positive cells, where TuJ-1-positive cells were not significantly affected (Figures 5D–G), which is consistent with the results of RSL3 treatment.

These results illustrated that PM were the most susceptible to ferroptosis in both monoculture and tri-culture systems. And it also demonstrated that the tri-culture system was more resistant to ferroptosis than that in the monoculture (for example, the 2.5 μM RSL3 yielded 50% PA and 77% PM in the monoculture, respectively, Figures 1F–H; vs. 22% PA and 68% PM in the tri-culture system, Figures 4E–H). Specifically, a small but significant proportion of neurons undergoing ferroptosis (about 20% death with 2.5 μM RSL3 Figures 1F–H), where no neuronal death was detected in the tri-culture system, indicating that the tri-culture system may therefore protect neurons when stressed by ferroptosis, which is of clinical relevance.

## Discussion

Collectively, our results here indicate that ferroptosis may affect brain cells differentially among PA, PM, and PN, compared to apoptosis and autophagy. PM were the most susceptible to ferroptosis in both monoculture and tri-culture systems. These results were consistent with that microglia possessed the highest susceptibility to ferroptosis in a human iPSC-derived tri-culture system which consists of a relative percentage of astrocytes, microglia, and neurons (Ryan et al., 2021).

Apoptosis, autophagy, and ferroptosis share common regulatory elements, and the cross-talk of cell death have reported in neurological diseases (Dang et al., 2022). Here we found that ferroptosis affect brain cells differentially compared to apoptosis and autophagy *in vitro*, which may be caused by the different iron content among these brain cells since neither apoptosis nor autophagy has been reported to be affected by iron levels (Reinert et al., 2019). During the disease progression, the cells may respond to various stress differently, as previously demonstrated in the pathogenesis of stroke (Naito et al., 2020). It is also possible that brain cells from other brain regions act differently than the cortical cells we observed here, depending on their ability to handle iron stress. For example, we have found that dopaminergic neurons in substantial nigra were both iron and dopamine rich, compared to the adjacent ventral tegmental area, which was susceptible to 6-hydroxydopamine (6-OHDA) toxicity modeling PD (Hare et al., 2014).

Lipid peroxidation has been considered one of the most pivotal indicators of ferroptosis (Wenzel et al., 2017). Here we found significant but similar increases in lipid ROS across all three brain cells, indicating that lipid ROS response to ferroptosis cannot be accounted for the differentiated cell susceptibilities. Further, we found that *Gpx4* was only reduced in PN treated with RSL3 which may be caused by the differences in sensitivity of different cells to Gpx4-regulate ferroptosis (Zou et al., 2019). Studies have shown that specific deletion of *Gpx4* in neurons was neonatal mortality, while conditional *Gpx4* deletion in mice resulted in neurodegeneration and astrogliosis (Seiler et al., 2008; Yoo et al., 2012), indicating different roles of Gpx4 in neurons and glial cells.

Iron was essential for the execution of ferroptosis. Iron regulatory genes were shown to affect ferroptosis sensitivity, and iron chelators prevent ferroptosis *in vivo* and *in vitro* (Dixon et al., 2012; Wang et al., 2017). Here, we identified that PA, PM, and PN acted differently facing the challenges of ferroptosis or iron. It was previously suggested that iron levels were different among the brain cells, where microglia are the most iron-rich cells (Reinert et al., 2019). Microglia may therefore exert as a guardian of ferroptosis within the neuronal system because they are prone to brain diseases with iron accumulation (Reinert et al., 2019; Guo et al., 2021). In support of this hypothesis, we found that microglia undergo ferroptotic cell death in the tri-culture system in a concentration where the other cells were still intact, and they responded to iron toxicity significantly. It would be of interest to test this hypothesis further in a condition of the disease since we have previously found that in EAE mice for MS, iron accumulation was a later event compared to the activation of ferroptosis in neurons (Luoqian et al., 2022). In addition, it is possible that direct contact in co-culture or tri-culture systems, in which cell-cell contact directly and cell-extracellular matrix interact can be signaled by soluble factors (Paschos et al., 2015), making all cells in the tri-culture system more viable as observed here.

We have found that RSL3 significantly reduced the *Fpn* expression in microglia while there was an increased level in neurons, and we have suggested that the altered levels of *Fpn* may explain the differentiated sensitivity to ferroptosis as we observed. *Fpn*, also known as *SCL40A1*, is the gene responsible for the expression of ferroportin which is the only identified iron exporter in mammals to date. Ferroportin can transport iron into the blood from iron storage cells and thus maintaining systemic iron homeostasis (McKie et al., 2000; Zhang et al., 2018), and it is coupled with ceruloplasmin or amyloid precursor protein to export iron in brain cells (Lei et al., 2021). Erastin was suggested to induce ferroptosis with increased iron by regulating ferroportin in human breast carcinoma cells and endometriosis (Ouyang et al., 2020; Li et al., 2021). Knockdown of *Fpn* accelerated erastin-induced ferroptosis in neuroblastoma cells (Geng et al., 2018). Overexpression of *Fpn* in the hippocampus ameliorated ferroptosis in an AD mouse model (Bao et al., 2021). Therefore we speculated here that ferroportin changes observed protected neurons from ferroptotic stress, and the sensitivity of brain cells to ferroptosis may depend on their abilities to regulate iron.

Microglia are the immune-resident cells of the CNS and comprise a major link between inflammation and neurodegeneration. The cells can sensitize to inflammation and trigger neuroinflammatory responses (Borst et al., 2021). Recently, a new subset of disease-associated microglia (DAM) was identified, which may be therapeutically targeted (Deczkowska et al., 2018). Previous studies have suggested that microglia contributed to MS in part owing to its sensitivity to iron (Lee et al., 2019; Absinta et al., 2021), and we have previously demonstrated that ferroptosis inhibition or knockdown *Acsl4* improved the MS behavioral phenotypes, with reduced neuroinflammation and neuronal death in the EAE mice (Luoqian et al., 2022). In the current study, we found that microglia were the most sensitive to ferroptosis or iron toxicity. Therefore, targeting microglia, especially DAM, may provide a new therapeutic approach for neurological disorders with inflammation and immune defects.

## Conclusion

In conclusion, our findings highlight the possibility that brain cells may be individually targeted in disease since they respond to ferroptotic stress differently. Most of the current therapeutic strategies related to ferroptosis are to inhibit its occurrence, and it would be of critical importance to select the responsible cells to target. It also has implications in brain cancer therapy, where the dosage of ferroptosis inducers can affect the targeted cell types as we demonstrated here. The essential roles of microglia in maintaining brain homeostasis and functions, especially the role during cell death, may provide further clues on the selective cell death occurring in neurological diseases.

## Data availability statement

The original contributions presented in this study are included in the article/supplementary material, further inquiries can be directed to the corresponding author/s.

## Ethics statement

This animal study was reviewed and approved by the Institutional Guidelines of the Animal Care and Use Committee (K2018071, Sichuan University, China).

## Author contributions

PL and XSL: concept, design experiments, and project supervision. LJ, XLL, and YL: conduct experiments, analyze data, and prepare figures and manuscript. JW, XD, and HX: analyze data and prepare figures. All authors assisted with the maintenance of the availability of reagents, sample processing, and data analysis, and contributed to the editing of the manuscript and agreed to be accountable for the content of the work.
